# Evaluation of *Curcuma xanthorrhiza* Roxb. extract as a functional alternative to antibiotic growth promoters in broiler chicken nutrition

**DOI:** 10.14202/vetworld.2025.1944-1954

**Published:** 2025-07-17

**Authors:** Arnold Parlindungan Sinurat, Tuti Haryati, Maijon Purba, Tiurma Pasaribu, Yan Irawan, Ryan Haryo Setyawan, Ferdy Saputra, Muhammad Ilyas, Nila Miraya, Sumiati Sumiati

**Affiliations:** 1 Research Center for Animal Husbandry, Research Organization for Agriculture and Food, National Research and Innovation Agency, Cibinong, Bogor District 16915, West Java, Indonesia; 2 Research Center for Chemistry, Research Organization for Nanotechnology and Material, National Research and Innovation Agency, Serpong, Tangerang Selatan 15314, Banten, Indonesia; 3 Research Center for Applied Microbiology, Research Organization of Life Sciences and Environment, National Research and Innovation Agency, Cibinong, Bogor District 16915, West Java, Indonesia; 4 Research Center for Biosystematics and Evolution, Research Organization of Life Sciences and Environment, National Research and Innovation Agency, Cibinong, Bogor District 16915, West Java, Indonesia; 5 Department of Nutrition and Feed Technology, Faculty of Animal Science, IPB University, Jl. Agatis, Dramaga Bogor, West Java, Indonesia

**Keywords:** antibiotic alternatives, broiler performance, *Curcuma xanthorrhiza* Roxb, feed efficiency, ileal microbiota, phytogenic additive

## Abstract

**Background and Aim:**

The global ban on antibiotic growth promoters (AGPs) in poultry production has intensified the search for effective phytogenic alternatives. *Curcuma xanthorrhiza* Roxb., commonly known as Javanese turmeric, exhibits antimicrobial and antioxidant properties attributed to its bioactive compounds, including xanthorrhizol and curcumin. This study evaluated the potential of a novel adjuvant extract (adjuvant *C. xanthorrhiza* Roxb. [ACX]) derived from *C. xanthorrhiza* Roxb. to replace AGPs in broiler diets. This study aimed to assess the *in vitro* antimicrobial and antioxidant activities of ACX and determine its efficacy as a growth-promoting feed additive in broiler chickens relative to AGPs.

**Materials and Methods:**

ACX was produced through double extraction of dried *C. xanthorrhiza* rhizomes and standardized for xanthorrhizol and curcuminoids using high-performance liquid chromatography. *In vitro* assays determined the minimum inhibitory concentration (MIC) and minimum fungicidal concentration against *Escherichia coli* and *Aspergillus flavus*, and antioxidant activity was evaluated using a 2,2-diphenyl-1-picrylhydrazyl assay. A total of 420 Cobb CP707 broilers were allocated to seven dietary treatments, including a negative control, a virginiamycin-positive control, and five graded ACX concentrations (20–320 ppm). Growth performance, carcass traits, internal organ weights, digestive tract dimensions, and ileal microbiota were assessed over a 35-day feeding trial.

**Results:**

ACX demonstrated antimicrobial activity (MIC = 80 μg/mL) and antioxidant potential (half-maximal inhibitory concentration = 57.3 μg/mL). Supplementing with 20 ppm ACX increased body weight by 7% and improved feed conversion ratio by 8% compared to control birds. Unlike AGPs, ACX supplementation did not increase feed intake, suggesting enhanced nutrient utilization. ACX also reduced abdominal fat and liver weight, with minimal impact on carcass yield or immune-related organs. Microbiota analysis revealed increased abundance of Firmicutes and decreased abundance of Proteobacteria in ACX- and AGP-fed groups, without disrupting microbial diversity.

**Conclusion:**

ACX supplementation at 20 ppm effectively enhanced broiler performance, reduced fat deposition, and modulated gut microbiota, offering a promising phytogenic alternative to AGPs. These findings support the integration of *C. xanthorrhiza* Roxb. extract into poultry nutrition strategies aimed at sustainable production.

## INTRODUCTION

Poultry producers are continually exploring innovative strategies to enhance productivity and optimize flock performance. A widely adopted appr-oach has involved the use of non-nutritive feed add-itives, particularly antibiotic growth promoters (AGPs). However, a growing body of evidence has raised sign-ificant concerns regarding this practice, primarily due to the role of AGPs in promoting the emergence of antibiotic-resistant microorganisms, which pose a major public health hazard [1, 2]. In response, several countries, including Indonesia, have implemented regu-latory bans on the use of antibiotics in animal feed, thereby prompting a shift toward identifying alternative growth-promoting agents.

Among the promising alternatives, medicinal plants have garnered attention due to their longstanding use as sources of bioactive phytochemicals with pharma-cological efficacy [3]. One such plant, Javanese turmeric (*Curcuma xanthorrhiza* Roxb.), has been extensively studied for its potential application in animal nutrition. The rhizome of *C. xanthorrhiza* is known to contain approximately 48 phytochemical constituents, pri-marily terpenoids and curcuminoids, with xanthorrhizol and curcumin recognized as the dominant bioactive compounds [4]. These compounds have demonstrated substantial antimicrobial and antioxidant activities in various *in vitro* and *in vivo* studies [5–8], suggesting the feasibility of employing Javanese turmeric extract as a natural alternative to AGPs in poultry diets.

Previous attempts to utilize *C. xanthorrhiza* as a feed additive have yielded inconsistent results. For example, the inclusion of 200 mg/kg or 400 mg/kg of its essential oil in broiler diets did not significantly influence performance outcomes [9]. In contrast, administering 0.75 g/L of powdered turmeric through drinking water was shown to enhance body weight gain and improve the feed conversion ratio (FCR) in broilers [10]. These discrepancies may stem from variability in the standardization and quantification of active compounds within turmeric-based preparations. Such variability is influenced by multiple factors, including genotypic differences, harvest maturity, environmental growing conditions, and the specific extraction methods employed [4, 11].

Despite increasing interest in phytogenic feed additives as sustainable alternatives to AGPs, the scientific evidence surrounding their efficacy remains inconclusive. In the context of *C. xanthorrhiza* Roxb., a medicinal plant with recognized antimicrobial and antioxidant properties, studies have reported incon-sistent outcomes when applied to poultry production systems. Previous investigations have employed diverse forms and dosages of *C. xanthorrhiza*, ranging from crude powders and essential oils to aqueous extracts, without adequate standardization of bioactive compound concentrations. These methodological inconsistencies, coupled with variations in plant chemotype, harvest maturity, and extraction procedures, have contributed to variability in biological efficacy. Moreover, most studies have not linked *in vitr*o antimicrobial potency with *in viv*o performance outcomes or microbiota modulation in broilers. Importantly, no published research to date has evaluated a standardized extract of *C. xanthorrhiza* prepared using a dual-extraction technique nor has it systematically examined its im- pact on intestinal microbial communities using high-throughput sequencing technologies. Therefore, a comprehensive investigation integrating phytochemical profiling, *in vitr*o bioactivity assays, and *in viv*o phy- siological, microbiological, and performance metrics is critically needed to substantiate the utility of *C. xanth-orrhiza* as a functional feed additive.

The present study aimed to evaluate the efficacy of a novel adjuvant (adjuvant *C. xanthorrhiza* Roxb. [ACX]) derived from *C. xanthorrhiza* Roxb. as an alternative to conventional AGPs in broiler diets. Specifically, the study sought to (1) characterize the phytochemical composition of ACX using high-performance liquid chromatography (HPLC); (2) determine its antibacterial, antifungal, and antioxidant activities through minimum inhibitory concentration (MIC), minimum bactericidal/fungicidal concentration (MBC/MFC), and 2,2-diphenyl-1-picrylhydrazyl (DPPH) assays; (3) assess its effects on broiler growth performance, carcass traits, organ development, and feed efficiency during both starter and finisher phases; and (4) evaluate its influence on ileal microbiota composition using 16S ribosomal RNA (16S rRNA) amplicon sequencing. By integrating these multi-dimensional analyses, the study aimed to provide empirical evidence on the potential of ACX to serve as a scientifically validated and sustainable alternative to AGPs in modern poultry production systems.

## MATERIALS AND METHODS

### Ethical approval

The study protocol was reviewed and approved by the Animal Care and Use Division of the Ethical Clearance and Research Permit Commission of National Research and Innovation Agency (Approval No: 133/KE.02/SK/06/2023).

### Study period and location

The extraction of *Curcuma xanthorrhiza* was conducted at the Laboratory of the Research Centre for Chemistry, National Research and Innovation Agency, in Serpong, Banten between June and August 2023. The feeding trial was conducted at the Poultry Research Farm, Faculty of Animal Science, IPB University (Bogor), from September 12 to October 17, 2023.

### Preparation of ACX

The adjuvant ACX was prepared by extracting dried rhizomes of *C. xanthorrhiza* Roxb., following the method of Hwang *et al*. [12]. Briefly, 22 kg of dried rhizomes (90% dry matter) purchased from Magelang, Central Java, were pulverized using an 80–100 mesh sieve. The resulting powder was macerated in 70% methanol at a 1:10 (w/v) ratio for 24 h at 25^o^C-29^o^C (room temperature). The suspension was percolated for 4 h at 50°C and filtered through Whatman No. 4 filter paper. The filtrate was concentrated under vacuum (−0.8 bar, 50°C–60°C; Buchi R-250, Switzerland) and then dried at 50°C to yield 450 g of the adjuvant.

### Phytochemical analysis of ACX

Quantification of major phytochemicals in ACX was conducted using HPLC at the Biofarmaka Laborat-ory, IPB University. The final ACX preparation contained 76.93 mg/g xanthorrhizol, 3.57 mg/g bisdesmethoxy-curcumin, 55.68 mg/g demethoxycurcumin, and 159.11 mg/g curcumin.

### Antimicrobial activity assay

The antibacterial and antifungal activities of ACX were evaluated using microdilution methods to determine the MIC, MBC, and MFC. The tests were conducted in duplicate against *Escherichia coli* InaCC B-5 and *Aspergillus flavus* LL.06-F003.

- Microbial culturing: *E. coli* was cultured on Nutrient Agar (NA; Merck, Darmstadt, Germany) at 37°C for 24 h, and *A. flavus* was grown on Potato Dextrose Agar (PDA; Himedia, Mumbai, India) at room temperature for 5 days.
- Suspension preparation: Microbial colonies were suspended in 0.85% NaCl and diluted in Mueller Hinton Broth (MHB; Himedia) for bacteria and Potato Dextrose Broth (PDB; Himedia) for fungi. Tween 80 (Sigma-Aldrich, St. Louis, USA) was added for fungal suspensions.
- ACX and control preparations: ACX was dissolved in dimethyl sulfoxide (DMSO; Merck) and sterilized through 0.22 μm nylon syringe filtration (Himedia). Serial dilutions were prepared to achieve final concentrations of 0, 40, 80, 160, 320, 640, 1280, 2560, 5120, and 10240 μg/mL. Virginiamycin (Stafac-500, Phibro Animal Health Corporation, Teaneck, USA) was used as a positive control at 20–50 μg/mL.
- Assay conditions: The tests were conducted on 96-well plates. Bacterial cultures were incubated at 37°C for 24 h and fungal cultures at ~25°C for 48 h. Microbial growth was assessed by optical density (OD) at 620 nm using a Varioskan LUX multimode microplate reader (Thermo Fisher Scientific, Waltham, USA).
- MIC, MBC, and MFC determination: MIC was defined as the lowest concentration at which no visible OD increase was observed. MBC and MFC were determined by subculturing MIC-negative wells onto NA and PDA, respectively, and identifying the lowest concentration with 99%–99.5% killing activity.

### Antioxidant activity assay (DPPH method)

Antioxidant capacity was evaluated using the DPPH radical scavenging assay [13].

- Sample preparation: ACX stock solution was diluted to 32, 48, 64, 80, and 90 μg/mL in methanol (Merck). L-ascorbic acid (Sigma-Aldrich, Buchs, Switzerland) was prepared at 1–5 μg/mL as a positive control.
- Assay procedure: 0.2 mL of each dilution was mixed with 0.6 mL of 0.1 mM DPPH in methanol and incubated in the dark at room temperature for 30 min. Absorbance was read at 517 nm using the Varioskan LUX multimode microplate reader (Thermo Fisher Scientific).
- Calculation: Percent inhibition was calculated using the formula:

% inhibition = [(Abs_blank − Abs_sample) / Abs_blank] × 100

(Abs=Absorbance)

### Experimental design and animal management

A completely randomized design (CRD) feeding trial was conducted using 420 one-day-old Cobb CP707 broiler chicks, randomly allocated into 42 pens (10 birds/pen) across seven treatments and six replicates. The experiment was conducted at the Faculty of Animal Husbandry, IPB University, Bogor.

- Housing and feeding: Pens measured 1.0 m × 1.25 m and were equipped with rice husk bedding, a feeder, a drinker, and 70-W bulb heaters for the first 14 days. Chicks were vaccinated against ND and IBD at 3 and 10 days of age, respectively. Birds received feed and water *ad libitum*.
- Dietary treatments: Birds received one of seven treatments from days 1 to 35 in two feeding phases: Starter (days 1–14; 22% crude protein (CP), 2950 kcal/kg metabolizable energy (ME) and finisher (days 15–35; 20.5% CP, 3050 kcal/kg ME). The composition of the diets is presented in Table 1.
- Treatments included:
  - NC: Basal diet without additives
  - PC: NC + 20 ppm virginiamycin
  - ACX20: NC + 20 ppm ACX
  - ACX40: NC + 40 ppm ACX
  - ACX80: NC + 80 ppm ACX
  - ,ACX160: NC + 160 ppm ACX
  - ACX320: NC + 320 ppm ACX

**Table 1:** The composition and nutrient content of standard starter and grower diets.

Feed ingredients (%)	Starter (0–14 days old)	Grower (14–35 days old)
Yellow corn	60.34	61.16
Corn gluten meal	0.00	1.96
Rice bran	2.00	5.00
Soybean meal	28.35	22.53
Meat and bone meal	2.27	1.43
Fish meal	3.00	3.00
Crude palm oil	1.00	2.00
Dicalcium Phosphate	0.89	0.89
DL-methionine	0.38	0.38
L-lysine	0.28	0.28
Limestone	0.84	0.84
Salt	0.25	0.25
Vitamin mixture*	0.03	0.03
Mineral mixture**	0.05	0.05
Sodium bicarbonate	0.10	0.10
Choline chloride	0.10	0.10
Total	100.00	100.00
Nutrient contents (calculated values)		
Dry matter (%)	88.89	89.00
Crude fiber (%)	3.40	3.50
Metabolizable energy (kcal/kg)	2950	3050
Crude protein (%)	22.00	20.50
Crude fat (%)	4.56	5.84
Linoleic acid (%)	2.18	2.20
Calcium (%)	1.00	0.90
Available phosphorous (%)	0.50	0.456
Digestible lysine (%)	1.27	1.142
Digestible methionine (%)	0.667	0.670
Digestible methionine + cystine (%)	0.94	0.93
Digestible tryptophan (%)	0.222	0.196
Digestible threonine (%)	0.703	0.648

* Each kg contains Vitamin A 50,000,000 IU, Vitamin D3 9,000,000 IU, Vitamin E 80,000 mg, Vitamin K3 10,000 mg, Vitamin B1 10,000 mg, Vitamin B2 20,000 mg, Vitamin B6 12,000 Mg, Vitamin B12 100 mg, Vitamin C 10,000 mg, Ca-d-Pantothenate 40,000 mg, Nicotinamide 120,000 mg, Folic Acid 4,000 mg, and Biotin 100 mg;

** Each kg contains Iron (Fe) 4,000 mg, Copper (Cu) 720 mg, Mangan (Mn) 5,920 mg, Zinc (Zn) 3,600 mg, Cobalt (Co) 400 mg, Iodine (I) 40 mg, Selenium (Se) 14.40 mg

### Performance evaluation

Feed intake was calculated as the difference between feed offered and feed refused. Body weights were recorded at 0, 14, and 35 days. Mortality was recorded daily.

### Carcass and organ measurements

One bird per pen was sacrificed at day 35 to assess carcass yield, abdominal fat, and organ weights (liver, heart, thymus, bursa of Fabricius, and gizzard). Digestive tract lengths (duodenum, jejunum, and ileum) were measured. All organ weights were expressed relative to live body weight.

### Ileal microbial composition analysis

At 35 days, ileal contents from one bird per pen were collected into DNA/RNA Shield Fecal Collection Tubes (Zymo Research, USA). DNA was extracted using ZymoBIOMICS DNAKits (Zymo Research, Irvine, USA) and quantified using a NanoDrop 2000 (Thermo Fisher Scientific) and a 2% agarose gel electrophoresis.

- 16S rRNA sequencing: PCR amplification employed universal primers 338F and 806R [14]. Library preparation used the Native Barcoding Kit 24 V14 (Oxford Nanopore Technologies, Oxford, UK), and sequencing was conducted on a MinION device (Oxford Nanopore Technologies) with R10.4.1 (Oxford Nanopore Technologies) flow cells.
- Bioinformatics workflow: Sequences were proce-ssed using ShortRead and DADA2 in R 4.4.2 (https://cran.r-project.org/bin/windows/base/old/4.4.2/). Reads were filtered and assigned taxonomy using the Silva v138.1 (https://www.arb-silva.de/documentation/release-1381/) database. Visualization and taxonomic summaries were generated with tidyverse (https://www.tidyverse.org/) and SituSeq (https://github.com/jkzorz/Situ-Seq) [15]. Relative abundances were computed at the phylum level.

### Statistical analysis

Data were subjected to a one-way analysis of variance under a CRD with seven treatments and six replicates (pen as an experimental unit). Duncan’s multiple range test was applied when significant differences were observed (p < 0.05). Regression analysis assessed the linear and quadratic dose–response effects of ACX. Relative abundances of top bacterial phyla were compared using the Kruskal–Wallis test. All analyses were performed using R software version 4.4.2 https://cran.r-project.org/bin/windows/base/old/4.4.2/.

## RESULTS

### Antibacterial, antifungal, and antioxidant activities of ACX

Table 2 compares the antimicrobial and antifungal activities of the *C. xanthorrhiza* adjuvant (ACX) with virginiamycin (AGPs). AGPs exhibited approximately 1.6-fold stronger inhibitory activity against *E. coli* and *A. flavus* than ACX. The MIC of ACX against *E. coli* and its MFC against *A. flavus* were both recorded at 80 μg/mL, values comparable to those of AGPs.

**Table 2:** *In vitro* antimicrobial activity of the antibiotics and *Curcuma xanthorrhiza* extract.

Sample	*Escherichia coli*		*Aspergillus flavus*	
	
	MIC	MBC	MIC	MFC
ACX (μg/mL)	80	80	80	80
Virginiamycin (μg/mL)	>50	>50	>50	>50

MIC=Minimum inhibitory concentration, MBC=Minimum bactericidal concentration, MFC=Minimum fungicidal concentration

Table 3 presents the antioxidant activity of ACX as determined by the DPPH scavenging assay. The scavenging activity increased proportionally with ACX concentration. The IC_50_ of ACX was calculated as 57.3 μg/mL, categorizing it as an active antioxidant based on the standard classification (IC_50_ = 50–100 μg/mL).

**Table 3:** Antioxidant activity of *C. xanthorrhiza* extract.

Sample	Concentration (µg/mL)	% inhibition	IC_50_ (µg/mL)
*C. xanthorrhiza*	32	21.75	57.30
	48	52.96	
	64	63.15	
	80	65.38	
	90	69.21	
Ascorbic acid	1	4.92	4.36
	2	11.52	
	3	18.64	
	4	49.56	
	5	62.05	

*C. xanthorrhiza=Curcuma xanthorrhiza*, IC_50_=Half-maximal inhibitory concentration

### Influence of AGPs and ACX on broiler performance

#### Starter phase (days 1–14)

As shown in Table 4, there were no significant differences in feed intake, body weight gain, or survival rate among treatment groups (p > 0.05). However, significant differences were observed in FCR, where broilers receiving AGPs or higher doses of ACX achieved 7%–8% improved feed efficiency compared to the negative control (negative control). Regression analysis revealed a quadratic response of FCR to increasing ACX doses, with the optimal FCR observed at 160 ppm.

**Table 4:** Performances of broiler chickens fed with ACX adjuvant or AGPs.

Observation	NC	NC + antibiotic (AGPs)	Level of ACX adjuvant					p-value		
	
			20 ppm	40 ppm	80 ppm	160 ppm	320 ppm	Analysis of variance	Linear	Quadratic
Performance during the starter period (1–14 days)										
Day old cick body weight = g/b	38.3 ± 2.4	37.6 ± 2.9	37.6 ± 3.1	37.4 ± 3.2	37.6 ± 2.7	37.6 ± 2.7	37.7 ± 2.8	0.99	0.93	0.94
Feed intake (g/b/d)	30.2 ± 3.3	33.0 ± 2.2	31.0 ± 1.4	33.0 ± 1.3	31.6 ± 3.4	31.5 ± 1.9	31.1 ± 1.8	0.31	0.86	0.62
BW gain (g/b)	330.0 ± 32.5	376.6 ± 19.5	342.2 ± 13.8	352.6 ± 22.9	343.3 ± 31.7	349.4 ± 23.2	351.5 ± 26.5	0.1	0.28	0.44
FCR	1.336 ± 0.030^a^	1.231 ± 0.043^b^	1.268 ± 0.043^ab^	1.295 ± 0.060^ab^	1.279 ± 0.082^ab^	1.227 ± 0.083^b^	1.239 ± 0.043^b^	0.03	0.01	0.01
Viability, %	100.0 ± 0.0	98.3 ± 4.1	98.3 ± 4.1	100.0 ± 0.0	98.3 ± 4.1	100.0 ± 0.0	100.0 ± 0.0	0.68	0.41	0.71
Performance during the grower period (15–35 days)										
Feed intake (g/b/d)	99.9 ± 5.9	109.9 ± 5.3	100.8 ± 6.9	101.9 ± 3.4	103.9 ± 6.4	103.0 ± 8.9	99.9 ± 5.1	0.11	0.69	0.34
Body weight gain (g/b)	1182.3 ± 64.6^c^	1290.0 ± 37.1^a^	1276.1 ± 46.5^ab^	1232.5 ± 69.9^abc^	1213.6 ± 61.8^abc^	1201.1 ± 78.4^bc^	1176.9 ± 62.7^c^	0.02	0.11	0.28
FCR	1.774 ± 0.060^b^	1.790 ± 0.099^b^	1.657 ± 0.067^a^	1.739 ± 0.066^ab^	1.798 ± 0.077^b^	1.800 ± 0.099^b^	1.783 ± 0.048^b^	0.03	0.08	0.22
Viability, %	97 ± 5	97 ± 8	100 ± 0	100 ± 0	98 ± 0	100 ± 0	100 ± 0	0.47	0.31	0.60
Performance during the whole period (1–35 days)										
Feed intake (g/b/d)	72.0 ± 4.1	79.1 ± 2.5	72.8 ± 4.6	74.3 ± 2.1	75.0 ± 3.9	74.4 ± 5.8	72.4 ± 3.7	0.07	0.71	0.31
Body weight gain (g/b)	1512.3 ± 71.7^c^	1666.6 ± 45.1^a^	1618.3 ± 54.2^ab^	1585.1 ± 55.1^abc^	1556.9 ± 74.8^bc^	1550.5 ± 86.1^bc^	1528.4 ± 81.0^c^	<0.01	0.39	0.59
FCR	1.703 ± 0.093^a^	1.687 ± 0.110^a^	1.567 ± 0.053^b^	1.631 ± 0.043^ab^	1.683 ± 0.067^a^	1.652 ± 0.073^ab^	1.707 ± 0.071^a^	0.04	0.10	0.23
Viability, %	96.7 ± 5.2	95.0 ± 8.4	98.3 ± 4.1	100.0 ± 0.0	96.7 ± 5.2	100.0 ± 0.0	100.0 ± 0.0	0.31	0.16	0.31

BW=Bodyweight, FCR=Feed conversion ratio, ACX=Adjuvant *Curcuma xanthorrhiza* Roxb., AGP=Antibiotic growth promoters, NC=Negative control Different superscripts in the same column show significant differences (p<0.05)

#### Finisher phase (days 15–35)

During the finisher phase, both AGPs and ACX significantly improved body weight gain and reduced FCR (p < 0.05), although no significant differences in feed intake or survival were noted. The highest body weight and best FCR were recorded in broilers fed either AGPs or 20 ppm ACX. No clear dose-response trend in FCR was observed.

#### Overall growth period (days 1–35)

Across the full 35-day period, neither AGPs nor ACX had a significant effect on total feed intake or survival rate (p > 0.05). However, diets supplemented with AGPs or 20 ppm ACX resulted in faster growth (8%–9%) relative to the unsupplemented group. Notably, while AGPs increased growth through elevated feed intake (~10%), this was not evident in the 20 ppm ACX group. Consequently, birds in the 20 ppm ACX group achieved significantly superior FCR compared with both the control and AGP groups.

### Effects on carcass yield, breast meat, and abdominal fat

As detailed in Table 5, dietary treatments did not significantly influence carcass or breast meat yields (p > 0.05). However, differences in abdominal fat deposition were observed, with the highest levels recorded in the 40 ppm ACX group. Nonetheless, these differences were not statistically significant compared to the control or AGP-supplemented groups. No clear dose-dependent trend in the response of abdominal fat to ACX was evident.

**Table 5:** Carcass yield, breast meat, and abdominal fat of broiler chickens fed adjuvant *Curcuma xanthorrhiza* Roxb. adjuvant.

Treatments	Carcass, % BW	Breast meat (g/kg carcass)	Abdominal fat (g/kg carcass)
NC	69.4 ± 1.6	362.8 ± 24.4	16.0 ± 4.7^ab^
NC+antibiotic (AGPs)	69.0 ± 1.9	377.1 ± 13.2	14.5 ± 3.1^b^
NC+adjuvant (20 ppm)	70.5 ± 1.3	368.3 ± 29.7	14.5 ± 1.8^b^
NC+adjuvant (40 ppm)	68.4 ± 1.8	394.9 ± 26.3	19.8 ± 3.8^a^
NC+adjuvant (80 ppm)	70.4 ± 1.7	382.4 ± 13.4	11.8 ± 3.8^b^
NC+adjuvant (160 ppm)	70.7 ± 1.8	376.4 ± 14.9	14.2 ± 3.1^b^
NC+adjuvant (320 ppm)	70.7 ± 1.1	371.7 ± 31.3	14.1 ± 3.0^b^
p-value			
Analysis of variance	0.16	0.43	0.02
Linear	0.12	0.93	0.23
Quadratic	0.57	0.27	0.41

Different superscripts in the same column show significant differences (p < 0.05) BW=Bodyweight, NC=Negative control

### Effects on internal organ weights

Table 6 summarizes the effects of AGPs and ACX on the weights of internal organs. Liver and heart weights were significantly influenced by dietary treatments (p < 0.05), whereas the thymus and bursa of Fabricius were unaffected (p > 0.05). Liver weight showed a linear decrease with increasing ACX or AGP supplementation, with the lowest value observed in the 320 ppm ACX group. Only the 20 and 320 ppm ACX treatments exhibited statistically significant reductions compared to the control (p < 0.05). Heart weight displayed a quadratic dose response, peaked at 40 ppm ACX and declined at higher doses. The weights of the thymus and bursa of Fabricius did not vary significantly among treatments.

**Table 6:** Weight of some internal organs of broilers is affected by supplementation of AGPs or adjuvant *Curcuma xanthorrhiza* Roxb. (g/kg body weight).

Treatments	Liver	Heart	Thymus	Bursa of Fabricius
Negative control	21.6 ± 2.1^ab^	4.5 ± 0.4^ab^	2.53 ± 1.1	0.76 ± 0.4
Antibiotic (AGPs)	19.3 ± 2.8^abc^	4.0 ± 0.3^b^	2.93 ± 1.1	0.38 ± 0.1
Adjuvant 20 ppm	17.8 ± 1.4^c^	4.2 ± 0.3^b^	2.87 ± 0.7	0.47 ± 0.1
Adjuvant 40 ppm	22.6 ± 5.0^a^	5.0 ± 0.5^a^	2.41 ± 0.8	0.56 ± 0.2
Adjuvant 80 ppm	18.7 ± 1.2^bc^	4.3 ± 0.6^b^	3.26 ± 1.2	0.50 ± 0.2
Adjuvant 160 ppm	18.8 ± 2.8^bc^	4.9 ± 0.7^a^	2.76 ± 0.6	0.36 ± 0.1
Adjuvant (320 ppm)	17.2 ± 1.0^c^	4.1 ± 0.4^b^	2.56 ± 0.8	0.50 ± 0.2
p-value				
Analysis of variance	0.01	<0.01	0.67	0.07
Linear	0.02	0.27	0.76	0.16
Quadratic	0.80	0.03	0.33	0.04

Different superscripts in the same column show significant differences (p < 0.05)*.* AGP=Antibiotic growth promoters, ppm=Parts per million.

### Effects on digestive tract dimensions

Table 7 presents the dimensions of digestive org-ans across treatment groups. No significant differences were found in the weights or lengths of the gizzard, duodenum, jejunum, or ileum (p > 0.05), indicating that neither AGP nor ACX supplementation affected dige-stive tract morphology.

**Table 7:** Relative sizes of the digestive tracts of broilers fed with adjuvant as a replacer of the AGPs.

Treatments	Gizzard, g/kg BW	Duodenum, cm/kg BW	Jejunum, cm/kg BW	Ileum, cm/kg BW
Negative control	14.76 ± 1.64	7.85 ± 0.85	13.60 ± 2.17	10.45 ± 1.08
Antibiotic (AGPs)	13.89 ± 2.23	7.04 ± 0.72	12.49 ± 2.03	9.57 ± 1.33
Adjuvant 20 ppm	14.69 ± 1.73	7.95 ± 1.13	12.19 ± 2.02	10.39 ± 1.51
Adjuvant 40 ppm	14.27 ± 1.88	7.49 ± 2.38	13.12 ± 2.93	11.05 ± 2.18
Adjuvant 80 ppm	16.65 ± 2.47	8.38 ± 1.26	13.98 ± 1.14	10.28 ± 1.54
Adjuvant 160 ppm	15.60 ± 2.18	6.91 ± 1.09	13.36 ± 2.28	10.82 ± 0.95
Adjuvant (320 ppm)	16.57 ± 2.30	7.68 ± 0.93	11.89 ± 1.87	10.32 ± 1.51
p-value				
Analysis of variance	0.16	0.46	0.58	0.75
Linear	0.08	0.22	0.10	0.29
Quadratic	0.50	0.32	0.68	0.87

AGP=Antibiotic growth promoters, BW=Bodyweight

#### Relative abundance at the phylum level

**Figure 1:**
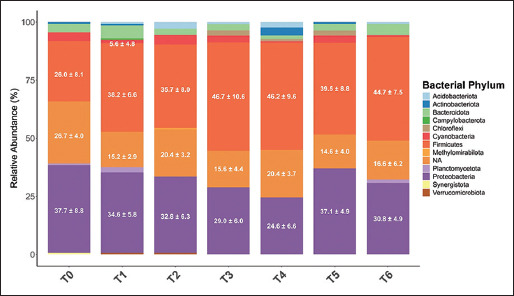
Microbiota in the ileal digesta of broilers fed the diet supplemented with AGPs or ACX. (T0 = Control [C]; T1 = C + AGPs; T2 = C + 20 ppm ACX; T3 = C + 40 ppm ACX; T4 = C + 80 ppm ACX; T5 = C + 160 ppm ACX; T6 = C + 320 ppm ACX). AGP=Antibiotic growth promoters, ACX=Adjuvant of *Curcuma xanthorrhiza* Roxb. Values: Mean + standard error of the mean (labeled for phyla >5% abundance).

The relative abundance of 12 bacterial phyla in ileal digesta is presented in Figure 1 and Table 8. Firmicutes (40.3%) and Proteobacteria (32.4%) were predominant in 35-day-old broilers. Supplementation with AGPs or ACX increased Firmicutes abundance compared to the control (26.00%), with the highest value (46.66%) observed in the 40 ppm ACX group. Conversely, Proteobacteria abundance decreased in response to supplementation, with the lowest prop-ortion (24.58%) detected in the 80 ppm ACX group and the highest in the control group (37.74%).

**Table 8:** Relative abundances of the most abundant phylums among all treatments.

Treatments	Firmicutes	Proteobacteria
Negative control	26.00 ± 19.72	37.74 ± 21.55
Antibiotic (AGPs)	38.19 ± 16.05	34.55 ± 14.26
Adjuvant 20 ppm	35.71 ± 19.62	32.78 ± 15.555
Adjuvant 40 ppm	46.66 ± 25.88	29.00 ± 14.60
Adjuvant 80 ppm	46.22 ± 23.51	24.58 ± 16.06
Adjuvant 160 ppm	39.50 ± 21.66	37.05 ± 12.10
Adjuvant (320 ppm)	44.71 ± 18.46	30.80 ± 11.91
p-value		
Analysis of variance	0.69	0.48
Linear	0.34	0.87
Quadratic	0.45	0.94

AGP=Antibiotic growth promoters

#### Microbial diversity and ordination analysis

**Figure 2:**
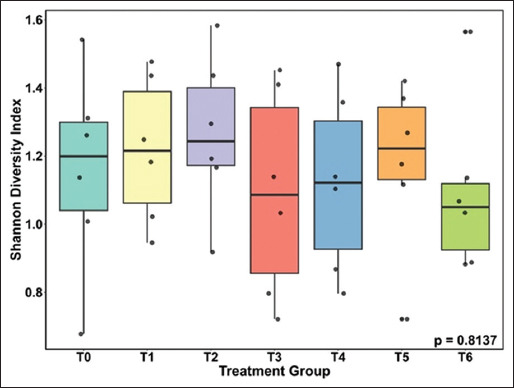
Shannon diversity index in the ileal digesta of broilers fed the diet supplemented with AGPs or ACX. (T0 = Control [C]; T1 = C + AGPs; T2 = C + 20 ppm ACX; T3 = C + 40 ppm ACX; T4 = C + 80 ppm ACX; T5 = C + 160 ppm ACX; T6 = C + 320 ppm ACX). AGP=Antibiotic growth promoters, ACX=Adjuvant of *Curcuma xanthorrhiza* Roxb.

Shannon diversity indices did not differ signi-ficantly among treatment groups (p = 0.8137; Figure 2). The narrow index range (0.8–1.2) across groups – including the NC (T0), antibiotic (AGP; T1), and adjuvant-supplemented groups (T2–T6) – indicates limited variability in microbial richness and evenness.

**Figure 3:**
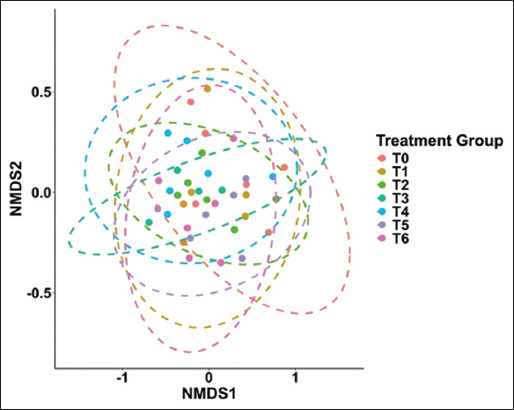
The non-metric multidimensional scaling plot based on Bray-Curtis dissimilarities in the ileal digesta of broilers fed the diet supplemented with AGPs or ACX. (T0 = Control [C]; T1 = C + AGPs; T2 = C + 20 ppm ACX; T3 = C + 40 ppm ACX; T4 = C + 80 ppm ACX; T5 = C + 160 ppm ACX; T6 = C + 320 ppm ACX). AGP=Antibiotic growth promoters, ACX=Adjuvant of *Curcuma xanthorrhiza* Roxb.

The non-metric multi-dimensional scaling (NMDS) ordination, based on Bray–Curtis dissimilarity, also sho-wed no distinct clustering by treatment group (Figure 3), supporting the findings of the Shannon index. The overlapping distribution along the NMDS1 axis (−1–1) suggests minimal divergence in overall community composition, indicating that neither AGP nor ACX supplementation, even at the highest dose, substantially altered beta diversity or microbiota structure.

## DISCUSSION

### Enhanced antimicrobial and antioxidant potency of ACX

The ACX extract demonstrated superior anti-microbial activity against *E. coli* compared to the previous studies, which reported MICs exceeding 500 μg/mL [16] or even 3200 μg/mL [17]. Remarkably, ACX was approximately 50% more potent against *E. coli* than isolated xanthorrhizol, a primary sesquiterpene in *C. xanthorrhiza*, which requires >128 μg/mL for inhibition [18]. Conversely, pure xanthorrhizol showed exceptional antifungal activity against *A. flavus*, with MIC and MFC values of 2 μg/mL and 4 μg/mL, respectively [19].

The antioxidant activity of ACX aligns with the findings of Widyastuti *et al*. [20], who reported half-maximal inhibitory concentration (IC_50_) values ranging from 32.53 to 77.19 μg/mL for the methanol extracts of *C. xanthorrhiza*. Variations in plant origin, extraction method, and solvent type contribute to these differences in antioxidant efficacy [21, 22].

### Novel application of ACX as a phytogenic adjuvant

Unlike earlier studies that used powdered or crude extracts of *C. xanthorrhiza* without consistently improving broiler performance, this study employed a refined adjuvant formulation of *C. xanthorrhiza* (ACX). The innovative application of ACX as a growth-promoting feed additive demonstrated effects comparable to AGPs.

During the starter phase, AGPs and high-dose ACX (160 or 320 ppm) improved FCRs by 8.2% and 7.9%, respectively, compared to the NC. However, low-dose ACX (20 ppm) was less effective in this phase, potentially due to its higher MIC (80 μg/mL) compared to virginiamycin (50 μg/mL), and to the immature immune system of broiler chicks during early life (6–13 days) [23].

### Superior efficacy of low-dose ACX in later growth phases

In contrast to the starter period, the finisher and overall growth periods showed superior performance with 20 ppm ACX supplementation, surpassing AGPs in FCR and body weight gain. While AGPs improved performance through increased feed intake (~10%), the 20 ppm ACX group achieved comparable or better outcomes with more efficient nutrient utilization.

The bioactive profile of ACX, which includes anti-microbial and antioxidant compounds, may explain its enhanced effect relative to AGPs. Antioxidant supplementation has been shown to enhance body weight and feed efficiency in broilers [24]. However, increasing ACX concentrations beyond 20 ppm showed a diminishing return, possibly due to mild dose-dependent toxicity, a phenomenon also observed in other plant-based bioactive supplements [25, 26].

### Comparison with previous phytogenic trials

Previous studies have yielded mixed results rega-rding the supplementation of *C. xanthorrhiza* in broilers. Doses of 375–1500 mg/kg turmeric powder (equivalent to 5.9–24 mg/kg xanthorrhizol and 7.5–30 mg/kg curcumin) had no significant impact on growth or nut-rient digestibility [27]. Similarly, essential oils and extracts containing 56–112 mg/kg or 81.2 mg/kg xanthorrhizol also failed to improve performance [9, 28]. Only water-based delivery (0.75 g/L) demonstrated notable benefits [10]. In this study, optimal results were achieved with 20 ppm ACX, delivering 1.5 mg xanthorrhizol, 3.2 mg curcumin, 1.1 mg demethoxycurcumin, and 0.1 mg bisdesmethoxycurcumin per kg of feed.

### Effect on carcass yield and fat deposition

Neither AGPs nor ACX significantly influenced carcass or breast meat yield. These findings are cons-istent with those of Li *et al*. [29], though the antibiotic types differed (chlortetracycline and tylosin). Notably, abdominal fat levels were reduced in broilers fed 20 ppm ACX, resulting in a 9.4% decrease compared to the control. This reduction may be attributed to curcumin’s capacity to suppress lipogenesis by reducing liver triglyceride concentrations [30], as well as the anti-adipogenic effects of *C. xanthorrhiza* observed in rodent models [31]. However, other studies reported by Akbarian *et al*. [28] and Hidanah *et al*. [32] have also reported inconsistent effects on fat deposition when using turmeric powders or extracts.

### Effect on digestive organ morphology

ACX and AGPs did not significantly affect the size or weight of digestive tract organs (gizzard and intestines), aligning with previous research by Hosseini *et al*. [9] and Akbarian *et al*. [28]. Morphological changes in digestive organs are typically associated with variations in feed efficiency. For example, Rougière *et al*. [33] found that improved FCR correlated with increased gizzard weight and reduced small intestine size. The lack of significant changes in this study suggests that perfor-mance improvements were unrelated to digestive tract anatomy.

### Influence on internal organs related to immunity and metabolism

Liver and heart weights were slightly reduced in the best-performing group (20 ppm ACX), whereas the thymus and bursa of Fabricius remained unaffected. The literature presents mixed outcomes: Some report no changes in immune organ weights with AGPs or phytogenic additives [25, 34], whereas others noted increased weights with AGPs or organic acids [35]. Results for *C. xanthorrhiza* have been similarly incon-sistent [9, 28, 36]. The observed reduction in liver and heart size with 20 ppm ACX could reflect an optimal metabolic or anti-inflammatory state indu-ced by xanth-orrhizol and curcumin, which are known for their hepat-oprotective and anti-inflammatory effects [37–39].

### Modulation of gut microbiota and mechanistic insights

The enhancement of Firmicutes abundance in the ileum following ACX supplementation mimics the effect of AGPs suggesting modulation of the gut microbiota as a potential mechanism of action. Firmicutes, parti-cularly *Lactobacillus*, dominate the gut of young bro-ilers [40]. Further, microbial profiling at the species level is warranted to elucidate specific bacterial shifts. The 20 ppm dose selection was informed by MIC data, aligning with the effective virginiamycin concentration in poultry diets [41].

### Economic implications of ACX supplementation

From a production standpoint, the use of 20 ppm ACX offers cost advantages. A cost-efficiency calculation, which involved multiplying the feed cost per kilogram by FCR and setting the control as the baseline (100%), revealed relative costs of 100% (control), 99.6% (AGP), and 92.5% (ACX). This analysis factored in AGP pricing (Rp. 1,260,000/kg), ACX pricing (Rp. 2,440,000/kg), and respective FCR values over the 35-day period (1.703, 1.687, and 1.567).

## CONCLUSION

The present study demonstrated that ACX, a novel adjuvant extract derived from *C. xanthorrhiza* Roxb., possesses potent antimicrobial and antioxidant properties, with an MIC of 80 μg/mL against *E. coli* and an IC_50_ of 57.3 μg/mL in the DPPH assay. When included in broiler diets, ACX significantly improved growth performance indicators such as body weight gain and FCR, with the 20 ppm dose producing the most notable effects. At this concentration, broilers exhibited a superior FCR (1.567) compared to the control (1.703) and virginiamycin group (1.687), alongside a reduction in abdominal fat and improved gut microbial balance without adversely affecting internal organ morphology or function.

These findings suggest that ACX may serve as an effective phytogenic alternative to AGPs in poultry production. Particularly, the 20 ppm dose of ACX offers practical advantages by improving feed efficiency and supporting gut health, which can reduce the industry’s dependence on AGPs without compromising productivity. The dual functional role of ACX, combining antimicrobial and antioxidant activities, makes it especially suitable for addressing common challenges in intensive broiler rearing, including pathogen pressure and oxidative stress.

The major strengths of this study include the use of a standardized ACX preparation with quantifiable phytochemical content, a comprehensive approach that integrates both *in vitro* and *in vivo* assessments, and an economic analysis demonstrating cost-effectiveness. However, some limitations should be noted. The study did not assess immune or oxidative stress biomarkers, and microbial community profiling was limited to the phylum level, thus overlooking species-specific dynamics. Furthermore, while low-dose ACX proved beneficial, the physiological impact of higher doses remains insufficiently characterized.

Future studies are warranted to investigate the immunological and molecular mechanisms by which ACX confers performance benefits. Evaluating gene expression, oxidative stress parameters, and gut barrier integrity could provide mechanistic insights. Addi- tionally, high-resolution microbiome analysis at the genus or species level would enhance our understanding of gut microbial shifts. Long-term trials in varied production environments and across different broiler breeds are essential to validate the scalability of these findings. The potential synergistic use of ACX with other feed additives, such as probiotics or enzymes, also merits investigation.

In conclusion, ACX at 20 ppm represents a promising, costefficient, and biologically active phyto-genic additive capable of replacing AGPs in broiler diets. By supporting optimal growth, feed utilization, and intestinal health without deleterious effects on vital organs, ACX offers a scientifically grounded solution for antibioticfree poultry production. With further mec-hanistic validation and field testing, ACX could play a central role in advancing sustainable and resilient poul-try nutrition strategies.

## AUTHORS’ CONTRIBUTIONS

APS: Conceptualization, investigation, visualiz-ation, data curation, formal analysis, and reviewed and edited the manuscript. TH, MP, and YI: Data curation, investigation, and validation. TP: Data curation, inves-tigation, validation, and reviewed and edited the man-uscript. RHS: Data curation, investigation, validation, and drafted the manuscript. FS: Data curation, formal analysis, software, investigation and, reviewed and edited the manuscript. MI and NM: Data curation and investigation. SS: Data curation and validation. All authors have read and approved the final manuscript.
